# Chronic Appendicitis—From Ambiguous Clinical Image to Inconclusive Imaging Studies

**DOI:** 10.3390/diagnostics12040818

**Published:** 2022-03-26

**Authors:** Agnieszka Brodzisz, Maryla Kuczyńska, Monika Zbroja, Weronika Cyranka, Czesław Cielecki, Magdalena Maria Woźniak

**Affiliations:** 1 Department of Pediatric Radiology, Medical University of Lublin, 20-059 Lublin, Poland; magdalena.wozniak@umlub.pl; 2 Department of Interventional Radiology and Neuroradiology, Medical University of Lublin, 20-059 Lublin, Poland; maryla.kuczynska@gmail.com; 3 Students’ Scientific Society at the Department of Pediatric Radiology, Medical University of Lublin, 20-059 Lublin, Poland; m.zbroja8888@gmail.com (M.Z.); weronika.cyranka@gmail.com (W.C.); 4 Department of Pediatric Surgery and Traumatology, Medical University of Lublin, 20-059 Lublin, Poland; czeslawcielecki@umlub.pl

**Keywords:** chronic appendicitis, acute abdomen, intestine, diagnostic imaging

## Abstract

A six-year-old boy visits a general practitioner due to diarrhea and abdominal pain with a moderate fever of up to 39 °C for 2 days. Treatment is initiated; however, the recurrence of abdominal pain is observed. Physical examination of the child at the emergency department reveals abdominal guarding and visible, palpable, painful intestinal loops in the left iliac and hypogastric regions—this is referred to as an ‘acute abdomen’. An X-ray shows single levels of air and fluid indicative of bowel obstruction. Ultrasound reveals distended, fluid-filled intestinal loops with diminished motility. The intestinal wall is swollen. Laboratory tests indicate increased inflammatory indices. Contrast-enhanced computed tomography examination of the abdominal cavity and lesser pelvis shows intestinal dilation. The loops were filled with liquid content and numerous collections of gas. The patient is qualified for a laparotomy. An intraoperative diagnosis of perforated gangrenous appendicitis with autoamputation was made. In addition, numerous interloop and pelvic abscesses, excessive adhesions, signs of small intestine micro-perforation, and diffuse peritonitis are found. The patient’s condition and laboratory parameters significantly improve during the following days of hospitalization. Despite the implementation of multidirectional, specialized diagnostics in the case of acute abdomen, in everyday practice we still encounter situations where the final diagnosis is made intraoperatively only.

## 1. Introduction

An acute abdomen is a medical condition that occurs unexpectedly, progresses rapidly and is commonly life-threatening. The underlying causes include diseases of the liver, stomach, intestines, and genitourinary system. Intestinal obstruction accounts for approximately 15% of all emergency visits for acute abdominal pain and is typically manifested by the following three symptoms: abdominal pain, vomiting, gas, and stool retention [1]. The exact cause of obstruction may not always be identified, but it can sometimes be accompanied by complicated appendicitis, some of which can be fatal. On the other hand, acute non-perforated appendicitis is one of the most common causes of intra-abdominal abscesses, with an estimated occurrence of up to 4.2% of patients [2]. Other complications include the following: perforation, which often leads to diffuse peritonitis, gangrene, or appendicular abscess [3]. In rare cases, the clinical image of appendicitis may be equivocal and pose diagnostic difficulties.

## 2. Case Presentation

**Figure 1:**
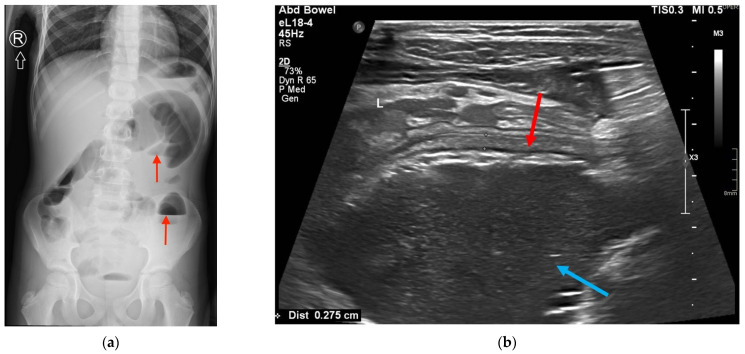
(**a**) Single air-fluid levels on X-ray (red arrows); (**b**) US examination showing intestinal wall edema (red arrow) and distended, fluid-filled lumen of the intestine (blue arrow).

A six-year-old boy visited a general practitioner (GP) due to diarrhea and abdominal pain with a moderate fever of up to 39 °C for 2 days. Based on a medical interview and physical examination, azithromycin was prescribed orally at a standard dose. The patient’s condition clearly improved. No imaging examinations were performed at that time. Four days following the end of treatment, the recurrence of abdominal pain, which was increased by changing the body’s position, was observed. The child was admitted to the emergency department where he was diagnosed with ‘acute abdomen’. The SARS-CoV19 antigen test was negative. Physical examination revealed abdominal guarding and visible, palpable, painful intestinal loops in the left iliac and hypogastric regions. A successful stool enema was conducted. Subsequently, diagnostic imaging was performed, including abdominal X-ray using Carestream Evolution Plus (Rochester, New York, NY, USA) and ultrasonography (US) using Philips Epiq 5 (Amsterdam, The Netherlands). Single air-fluid levels indicative of bowel obstruction were detected on X-ray (Figure 1a). The US revealed distended, fluid-filled intestinal loops with diminished motility. The intestinal wall was swollen (Figure 1b).

**Figure 2:**
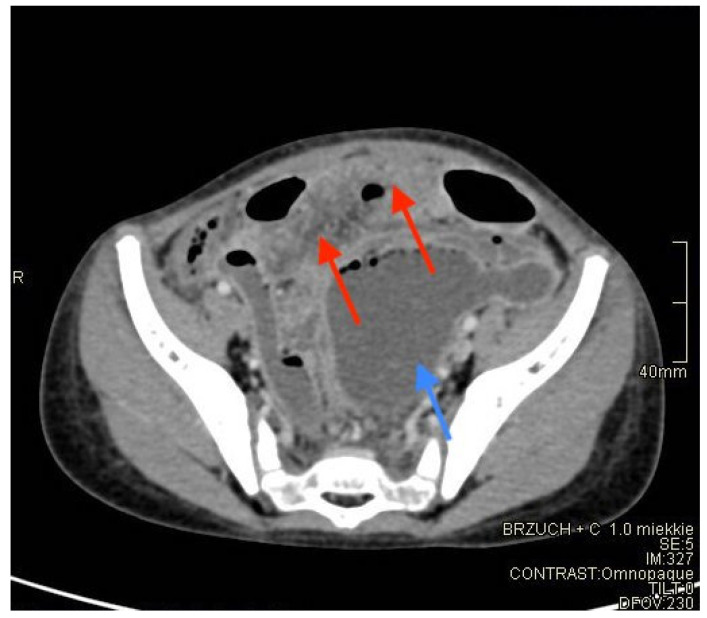
CT examination with contrast. Red arrow indicates mesenteric and omental fat stranding consistent with inflammatory infiltration and blue arrow—distended, fluid-filled lumen of the intestine.

The patient was referred to the Department of Pediatric Surgery and Traumatology. Due to developing sepsis and symptoms of multiple organ dysfunction syndrome (MODS) in the patient, an extensive panel of laboratory tests was performed, which indicated increased inflammatory indices such as PCT, CRP, ESR, ferritin, the level of leukocytes and neutrophiles (Table 1). It is worth emphasizing that PCT ≥ 10 ng/mL occurs almost exclusively in severe cases of septic shock [4]. It was decided to include abdominal computed tomography (CT) by Siemens Definition AS (Munich, Germany) in the diagnostic protocol due to alarming inflammatory parameters, inconclusive results of imaging studies, and diagnostic doubts. Contrast-enhanced CT examination of the abdominal cavity and lesser pelvis showed intestinal dilation of up to 55–60 mm, which was especially prominent within the sigmoid and descending colon. The loops were filled with liquid content (an average density of 17 HU) and numerous collections of gas along the intestinal wall. Loss of gut signature, together with mesenteric and omental fat stranding consistent with inflammatory infiltration, was seen in the middle abdominal region, left lower quadrant, and lesser pelvis (Figure 2).

Based on repeated physical examination, which revealed diffuse peritoneal signs, and all the previous laboratory and diagnostic imaging findings, the patient was qualified for surgical treatment, i.e., laparotomy. An intraoperative diagnosis of perforated gangrenous appendicitis with autoamputation was made. In addition, numerous interloops and pelvic abscesses, excessive adhesions, signs of small intestine micro-perforation, and diffuse peritonitis were found.

Although the stool culture was negative for Yersinia, the blood methicillin-resistant coagulase-negative Staphylococcus (MRCNS) epidermidis, and non-ESBL (extended-spectrum beta-lactamase)-producing Escherichia coli were detected in the blood and pus cultures, respectively.

**Figure 3:**
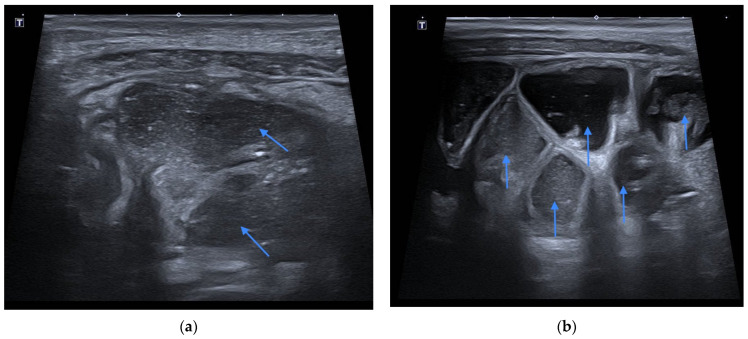
Fluid-filled intestinal loops with diminished motility in controlled US examination (**a**,**b**). Blue arrows show dilation of intestinal loop.

A follow-up US examination indicated mild edema of the bowel wall and peri-intestinal fat. Moderately dilated (lumen width approx. 15–20 mm), fluid-filled intestinal loops (of both small and large intestine) with diminished motility, were observed as well (Figure 3). The patient’s condition and laboratory parameters significantly improved during the following days of hospitalization. After another 4 days of consecutive observation the patient was discharged home in good condition.

## 3. Discussion

Diagnosis of appendicitis in children appears to be a well-known topic but may still be an issue due to non-specific clinical symptoms and variable, inconclusive imaging studies. Dilatation of bowel plexuses and obstruction require careful evaluation, and it is difficult to determine the cause. The incidence rate of chronic appendicitis is about 1.5% in patients who present with symptomatology similar to acute appendicitis [5]. In recent years, there have been only a few reports of severe diagnostic difficulties in appendicitis, mostly in adult patients. In one study, three-quarters of all patients with pain in the right lower quadrant but no serious signs of inflammation showed histologic criteria for chronic appendicitis [6]. Another study concerned two cases that completely differed in clinical presentation. They led the diagnosis to the following different possibilities: tropical infectious disease and possible malignancy; however, it turned out that, according to the radiological imaging, chronic appendicitis was diagnosed [7]. Furthermore, chronic appendicitis does not have well-defined criteria, which results in the fact that it is not considered by many specialists as a correct disease entity. Doctors from the Academic Medical Center in Amsterdam described the case of a patient who suffered from recurrent abdominal pain for 2 years, as well as nausea, vomiting, and fever up to 39 °C [8]. The man was primarily diagnosed with chronic or non-chronic infection, abscess, inflammatory bowel disease, and irritable bowel syndrome. However, after 2 years, the patient was finally diagnosed with recurrent appendicitis based on laparoscopic and histopathological examination. There are also remarkably few publications about the diagnostic difficulties of imaging studies in children. According to doctors from the College of Family Physicians of Canada, in patients with recurrent or chronic right lower quadrant pain and tenderness, chronic appendicitis should be considered, even despite a lack of characteristic laboratory findings [6]. Furthermore, the presented case report underlines that, apart from rare cases of unspecific clinical presentation of appendicitis, severe intestinal distension may disguise abscess formation on diagnostic imaging, leading to misinterpretation and wrong diagnosis. It should be kept in mind that in the pediatric patient, the clinical picture is often highly nonspecific and the course of acute abdominal conditions can be fatal. Thus, it is even more important to raise the sensitivity of the medical community to a detailed and careful analysis of conditions that imitate or provide a radiological picture of gastrointestinal obstruction or other diseases.

## 4. Conclusions

In summary, appendicitis is a condition that can be life-threatening if left untreated. The underlying inflammation may result in numerous complications and a significant deterioration of the patient’s health. Chronic appendicitis, especially in children, often has a non-specific image; therefore, detailed laboratory and imaging diagnostics are particularly important. In the above-described case of a six-year-old boy, clinical examination of the patient, ultrasound, and CT of the abdominal cavity suggested intestinal obstruction. However, based on the clinical and imaging diagnostics performed, its cause could not be determined. Only intraoperatively gangrenous perforated appendicitis with self-amputation, as well as multiple abscesses were diagnosed. Despite the implementation of multidirectional and specialized diagnostics in the case of acute abdomen, in everyday practice we still encounter situations where the final diagnosis is made intraoperatively only.

## Figures and Tables

**Table 1:** Laboratory indices of the patient.

Laboratory Indices	Value	Norm
CRP	18.34 mg/dL	<5 mg/dL
PCT	42.370 ng/mL	<0.05 ng/mL
ESR	53 mm/h	1–10 mm/h
Ferritin	1041 ng/mL	30–400 ng/mL
Leukocytosis	27,570/μL	4000–10,000/μL
Neutrophilia	22,730/μL	1800–8000/μL
Reduction of ATIII	59%	80–120%
D-dimers	6946 ng/mL	<500 ng/mL
NT-proBNP activity	1476 pg/mL	<125 pg/mL

Legend: CRP—C reactive protein. PCT—procalcitonin. ESR—erythrocyte sedimentation rate. ATIII—Antithrombin III. NT-proBNP—N-terminal pro hormone B-type natriuretic peptide.

## Data Availability

The data that support the findings of this study are available on reasonable request from the corresponding author. The data are not public available due to privacy.
